# Structures of the hydrate and dihydrate forms of the DNA-binding radioprotector methyl­pro­amine

**DOI:** 10.1107/S2056989018016791

**Published:** 2018-11-30

**Authors:** Jonathan Michael White, Samuel Charles Brydon, Thomas Fellowes

**Affiliations:** a School of Chemistry and BIO-21 Institute, University of Melbourne, Parkville, VIC 3010, Melbourne, Australia

**Keywords:** crystal structure, hydrates, hydrogen bonding, tautomerism

## Abstract

Methyl pro­amine {*N*,*N*,3-trimethyl-4-[6-(4-methyl­piperazin-1-yl)-1*H*,3′*H*-[2,5′-bibenzo[*d*]imidazol]-2′-yl]aniline}, C_28_H_35_N_7_O_2_, crystallized as both a dihydrate, C_28_H_31_N_7_·2H_2_O, and monohydrate, C_28_H_31_N_7_·H_2_O, form from water in the presence of β-cyclo­dextrin, in the *P*2_1_/*c* and *P*2_1_/*n* space groups, respectively. The two structures adopt different conformations and tautomeric forms as a result of the differing crystal packing as dictated by hydrogen-bonding inter­actions. The dihydrate crystallizes as a three-dimensional hydrogen-bonded network, while the monohydrate crystallizes as a two-dimensional hydrogen-bonded network.

## Abstract 2

The dihydrate and hydrate forms of the DNA-binding bis-benzimidazole radioprotector methyl­pro­amine are reported. These are the first single-crystal structures of bis-benzimidazoles related to Hoechst 33342 to be reported.

## Chemical context   

Methyl­pro­amine (1) is a bibenzimidazole derivative which binds in the minor groove of DNA in adenine-thymine-rich regions of four or more consecutive AT pairs (Martin *et al.*, 2004 ▸) and is related to the Hoechst family of DNA-binding bibenzimidazoles (Pjura *et al.*, 1987 ▸). Although the structure of methyl­pro­amine with the DNA dodeca­mer d(CGCGAATTCGCG)_2_ has been determined and reported by us, the structure of the free ligand has not yet been published as it is very difficult to obtain good quality crystals for these types of compounds. In order to examine the conformational and tautomeric differences between the uncomplexed ligand and that which is bound to DNA, the structures of both the dihydrate (1)·2H_2_O and the monohydrate (1)·H_2_O, which were grown from water in the presence of β-cyclo­dextrin, are reported.
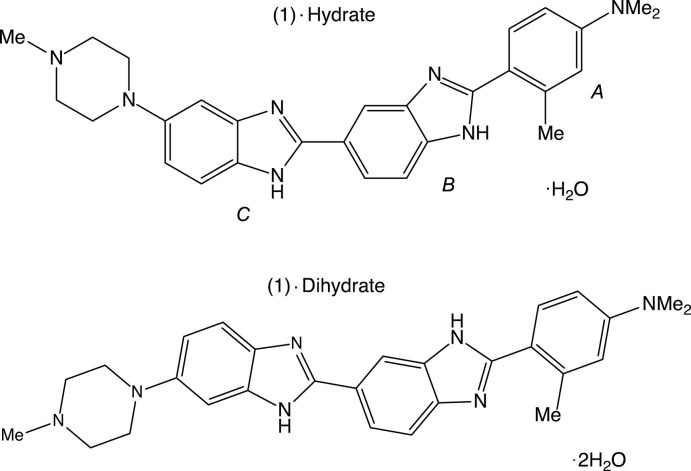


## Structural commentary   

**Figure 1:**
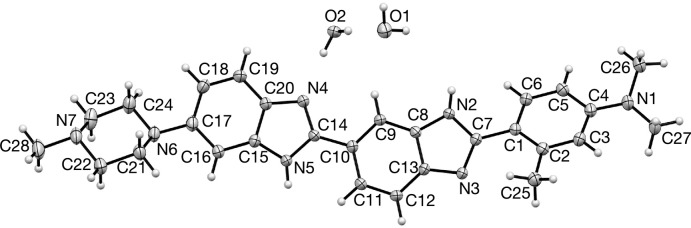
Displacement ellipsoid plot of the asymmetric unit for dihydrate (1)·2H_2_O.

Displacement ellipsoid plots for (1)·2H_2_O and (1)·H_2_O are presented in Figs. 1 ▸ and 2 ▸, respectively. The two structures represent two different conformations of (1); (1)·2H_2_O exists in an extended conformation as determined by the C9—C10—C14—N4 torsion angle which is 173.54 (14)° with an N1⋯N6 distance of 17.251 (2) Å while (1)·H_2_O adopts a crescent shape with a C9—C10—C14—N4 torsion angle of −19.8 (2)° and an N1⋯N6 distance of 16.859 (2) Å. In addition, they represent different tautomeric forms of (1); (1)·2H_2_O can be described as the N2, N4 tautomer whereas (1)·H_2_O exists in the crystal as the N2, N5 tautomer as defined by the numbering scheme used in Figs. 1 ▸ and 2 ▸. The tautomeric form adopted in each case is implied not only by the N—H hydrogen atoms, which were located in difference maps and refined satisfactorily without restraint, but also by the C—N bond distances of the two benzimidazole moieties within the structures (Tables 1 ▸ and 2 ▸). The tautomeric form assigned in each case is also supported by the inter­molecular hydrogen bonds that these N—H groups participate in. It is the inter­molecular hydrogen-bonded inter­actions involving these N—H groups which no doubt play a major role in which tautomer is adopted in each case in the solid state.

In both structures the *ortho*-methyl substituent in ring *A* lies on the opposite side of the structure to the N—H hydrogen atom of benzimidazole ring *B*, this is very likely for steric reasons; the dihedral angles between the two rings as defined by C2—C1—C7—N3 in (1)·2H_2_O and by C2—C1—C7—N2 in (1)·H_2_O, which are −30.0 (2) and −23.6 (2)°, respectively, reflect a balance between electronic effects which prefer coplanarity between the two aromatic rings and steric effects whereby the *ortho*-methyl group would be unreasonably close to the benzimidazole nitro­gen of ring *B*. The dihedral angles between the two benzimidazole rings (rings *B* and *C*) are −5.7 (2) and −19.8 (2)°, respectively.

The geometry of the *para*-di­methyl­amino substituent on ring *A* differs between the two structures; the mean C—N1—C angles are 116.4 and 119.7°, respectively, for (1)·2H_2_O and (1)·H_2_O, suggesting that the former is more pyramidalized, consistent with this are the significant differences in the C4—N1 bond distances which are 1.3923 (18) and 1.374 (2) Å for (1)·2H_2_O and (1)·H_2_O, respectively.

**Figure 3:**
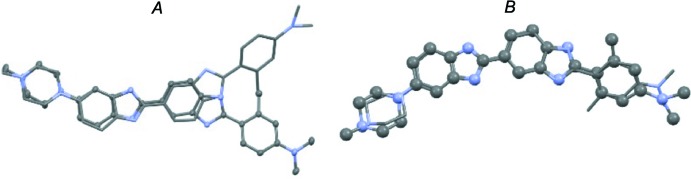
Overlay for the structures of *A*; (1)·2H_2_O and *B*; (1)·H_2_O with DNA-bound (1). In the LH-structure the DNA-bond ligand is indicated by capped sticks, while in the RH structure it is ball and stick.

It is inter­esting to compare the conformation of (1) in these two structures with that adopted by (1) when bound in the minor groove of the palindromic DNA dodeca­mer [d(CGCGAATTCGCG)_2_; Martin *et al.*, 2004 ▸]. The ligand must adopt the 2-H, 4-H tautomeric form with a crescent shape similar to that adopted by (1)·H_2_O so that it can direct the necessary N—H hydrogen-bond donors into the minor groove, in addition the *ortho*-methyl substituent on ring *A* must be facing away from the crescent. A superposition of the two structures with that of (1) bound to DNA is shown in Fig. 3 ▸.

## Supra­molecular features   

**Figure 4:**
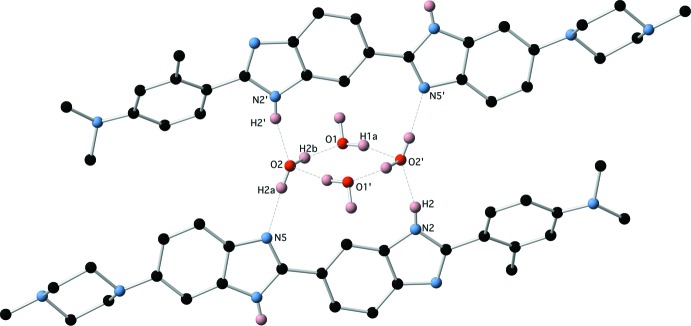
The water tetra­mer with diagonally hydrogen-bonded mol­ecules of (1).

**Figure 5:**
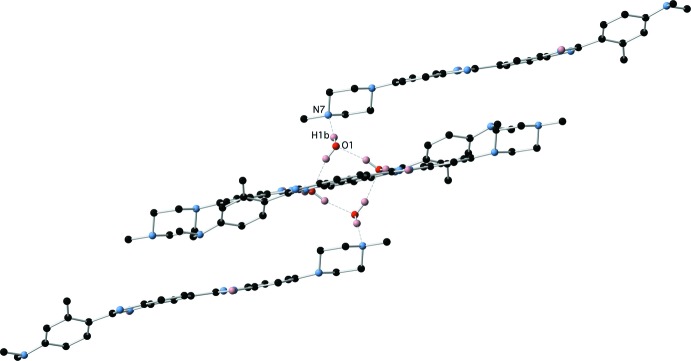
The water tetra­mer with additional hydrogen-bonded inter­actions with (1).

**Figure 6:**
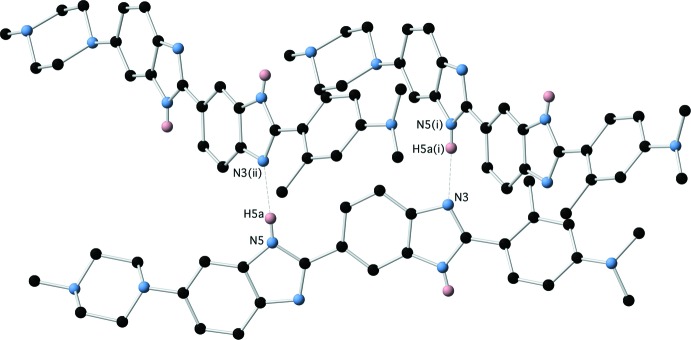
The cross-linking N—H⋯N hydrogen bonds between benzimidazole moieties mol­ecules of (1).

The structure of the dihydrate (1)·2H_2_O is characterized by the presence of a centrosymmetric water tetra­mer which provides a template around which the structure is built. This tetra­mer appears to be a common motif formed in crystalline hydrates with over 3689 examples of structures containing this motif in the Cambridge Structural Database [Version 1.23 update 5.39 (August 2018); Groom *et al.*, 2016 ▸]. The water tetra­mer is bridged across opposite diagonals by two mol­ecules of (1) by a combination of N—H⋯O and O—H⋯N hydrogen bonds involving the two benzimidazole groups (Fig. 4 ▸ and Table 3 ▸), the remaining O—H hydrogens form O—H⋯N hydrogen bonds to two further centrosymmetrically related mol­ecules of (1) via the tertiary piperazine nitro­gen N7 (Fig. 5 ▸ and Table 3 ▸). This cluster of four mol­ecules of (1) and the water tetra­mer is then extensively cross-linked by N—H⋯N hydrogen bonds between the remaining benzimidazole groups (Figs. 6 ▸ and 7 ▸ and Table 3 ▸).

**Figure 8:**
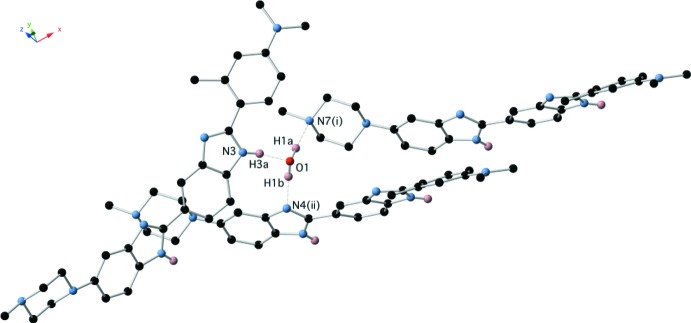
Hydrogen bonding between (1) and the water mol­ecule in (1)·H_2_O.

**Figure 9:**
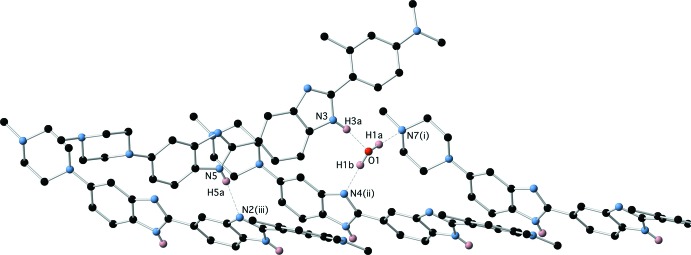
Direct N—H⋯N hydrogen bonds between benzimidazole moieties of (1)·H_2_O.

**Figure 10:**
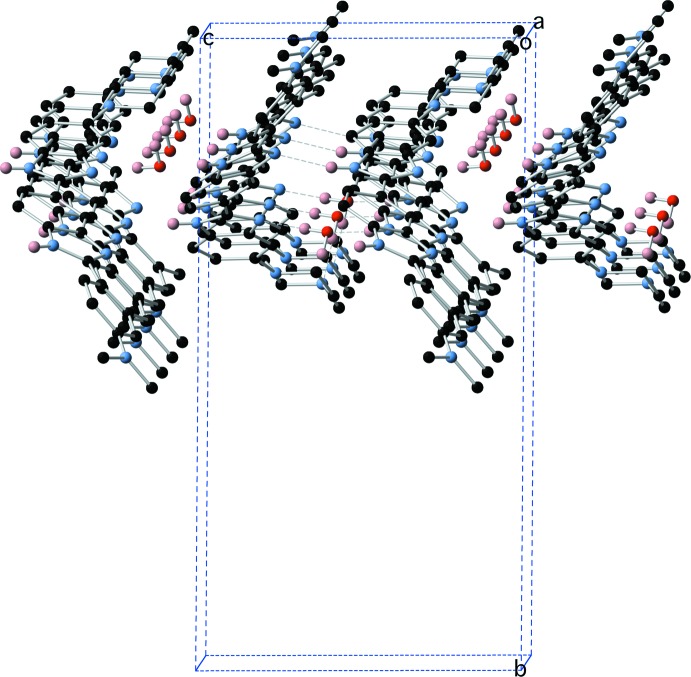
Two-dimensional hydrogen-bonded network in (1)·H_2_O.

The structure of the hydrate (1)·H_2_O is also characterized by extensive hydrogen-bonding inter­actions, both directly between the benzimidazole moieties of (1), and via the water mol­ecule. The water mol­ecule participates in two O—H⋯N hydrogen bonds as donor and one N—H⋯O hydrogen bond as acceptor to form a cluster of three mol­ecules of (1) (Fig. 8 ▸ and Table 4 ▸). This cluster is then further hydrogen bonded via N—H⋯N inter­actions between the remaining benzimidazole-based hydrogen-bond donors and acceptors (Fig. 9 ▸ and Table 4 ▸), to form two-dimensional hydrogen-bonded sheets lying in the (101) plane (Fig. 10 ▸).

## Database survey   

A search of the CSD (version 1.23; Groom *et al.*, 2016 ▸) for structures related to (1) uncovered no hits.

## Synthesis and crystallization   

The synthesis of methyl­pro­amine (1) has been previously reported (Martin *et al.*, 2004 ▸) but previous attempts to obtain crystals of the free ligand of suitable quality for X-ray analysis were not successful. In this study, crystals were serendipidously obtained during an attempt to obtain crystals of (1) complexed to β-cyclo­dextrin. Thus a solution of (1) (6.8mg) in 1 ml of water saturated with β-cyclo­dextrin was left in a vapour diffusion tank with acetone allowed to diffuse into the solution. It is worth noting that (1) has very low solubility in water in the absence of β-cyclo­dextrin. After 12 h, brown plates of (1) as its dihydrate developed, which were then harvested for X-ray analysis. The resulting solution when left to evaporate over a period of several months gave further needle-like crystals in a viscous matrix of β-cyclo­dextrin that were shown to be the monohydrate (1)·H_2_O.

## Refinement   

Crystal data, data collection and structure refinement details for (1)·2H_2_O and (1)·H_2_O are summarized in Table 5 ▸. In both structures, carbon-bound H atoms were placed in calculated positions and refined using a riding model, with methyl C—H = 0.96 Å and aromatic C—H = 0.93 Å and *U*_iso_(H) =1.5*U*_eq_(C) for methyl and 1.2*U*_eq_(C) for aromatic C—H. Hydrogen atoms attached to N and O were located in difference maps and allowed to refine with isotropic displacement parameters. In the structure of (1)·H_2_O there are solvent-accessible voids of 154 Å^3^ per unit cell; however, there was no significant difference electron density associated with these voids. The largest difference electron density of 0.5 e Å^3^ was associated with the piperazine group. Application of the SQUEEZE procedure (Spek, 2015 ▸) found eight electrons associated with the voids.

## Supplementary Material

Crystal structure: contains datablock(s) 1_dihydrate, 1_hydrate. DOI: 10.1107/S2056989018016791/sj5567sup1.cif

Structure factors: contains datablock(s) 1_dihydrate. DOI: 10.1107/S2056989018016791/sj55671_dihydratesup2.hkl

Structure factors: contains datablock(s) 1_hydrate. DOI: 10.1107/S2056989018016791/sj55671_hydratesup3.hkl

Supporting information file. DOI: 10.1107/S2056989018016791/sj55671_dihydratesup4.cml

Supporting information file. DOI: 10.1107/S2056989018016791/sj55671_hydratesup5.cml

CCDC references: 1881120, 1881119

Additional supporting information:  crystallographic information; 3D view; checkCIF report

## Figures and Tables

**Figure 2:**
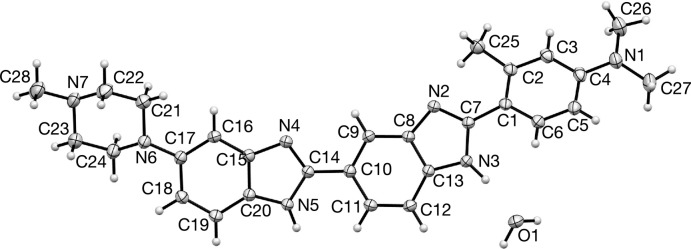
Displacement ellipsoid plot of the asymmetric unit for hydrate (1)·H_2_O.

**Figure 7:**
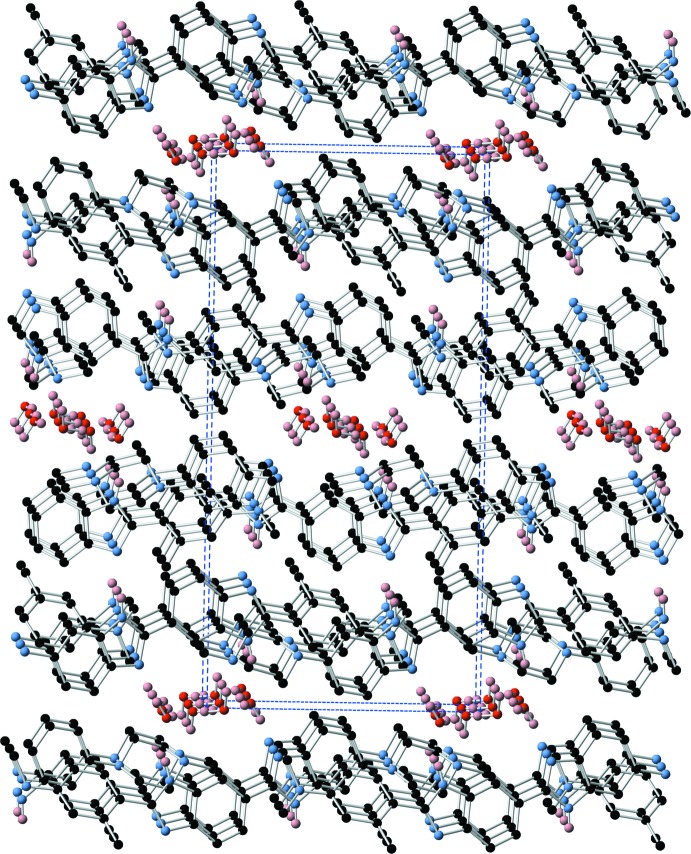
Three-dimensional hydrogen-bonded network in (1)·2H_2_O.

**Table 1:** Selected geometric parameters (Å, °) for (1)·2H_2_O[Chem scheme1]

C1—C7	1.4698 (18)	C10—C14	1.4701 (19)
C4—N1	1.3923 (18)	C14—N4	1.3708 (18)
C7—N2	1.3695 (17)	C14—N5	1.3215 (18)
C7—N3	1.3325 (18)	C17—N6	1.4321 (19)
			
C4—N1—C27	118.18 (13)	C17—N6—C24	114.80 (13)
C4—N1—C26	118.60 (13)	C17—N6—C21	113.32 (12)
C27—N1—C26	112.78 (13)	C24—N6—C21	109.48 (12)
			
C2—C1—C7—N3	−30.0 (2)	C9—C10—C14—N4	173.54 (14)
C6—C1—C7—N3	147.26 (15)	C3—C4—N1—C27	1.2 (2)
C2—C1—C7—N2	155.44 (14)	C5—C4—N1—C26	40.9 (2)
C6—C1—C7—N2	−27.3 (2)	C16—C17—N6—C24	−170.61 (14)
C9—C10—C14—N5	−5.7 (2)	C16—C17—N6—C21	62.58 (19)

**Table 2:** Selected geometric parameters (Å, °) for (1)·H_2_O[Chem scheme1]

C1—C7	1.468 (2)	C10—C14	1.464 (2)
C4—N1	1.374 (2)	C14—N4	1.3286 (19)
C7—N2	1.3344 (19)	C14—N5	1.3691 (19)
C7—N3	1.3687 (19)	C17—N6	1.4190 (19)
			
C4—N1—C27	120.55 (15)	C17—N6—C21	117.43 (13)
C4—N1—C26	119.35 (15)	C17—N6—C24	115.25 (13)
C27—N1—C26	119.08 (14)	C21—N6—C24	109.98 (13)
			
C2—C1—C7—N2	−23.6 (2)	C9—C10—C14—N5	161.50 (14)
C2—C1—C7—N3	154.23 (14)	C16—C17—N6—C21	3.4 (2)
C9—C10—C14—N4	−19.8 (2)	C18—C17—N6—C24	−48.47 (19)

**Table 3:** Hydrogen-bond geometry (Å, °) for (1)·2H_2_O[Chem scheme1]

*D*—H⋯*A*	*D*—H	H⋯*A*	*D*⋯*A*	*D*—H⋯*A*
C23—H23*B*⋯N2^i^	0.99	2.65	3.542 (2)	149
O1—H1*A*⋯O2^ii^	0.91 (3)	1.98 (3)	2.8665 (17)	164 (2)
O1—H1*B*⋯N7^iii^	0.94 (3)	1.91 (3)	2.8482 (18)	170 (3)
N2—H2⋯O2^ii^	0.856 (18)	1.944 (18)	2.7797 (15)	165.0 (17)
O2—H2*A*⋯N5	0.86 (2)	1.91 (2)	2.7685 (16)	175 (2)
O2—H2*B*⋯O1	0.90 (3)	1.86 (3)	2.7537 (18)	168 (3)
N4—H4*A*⋯N3^iv^	0.870 (19)	2.072 (19)	2.9411 (16)	176.7 (17)

Symmetry codes: (i) 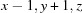; (ii) 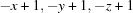; (iii) 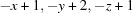; (iv) 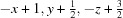.

**Table 4:** Hydrogen-bond geometry (Å, °) for (1)·H_2_O[Chem scheme1]

*D*—H⋯*A*	*D*—H	H⋯*A*	*D*⋯*A*	*D*—H⋯*A*
O1—H1*A*⋯N7^i^	0.93 (4)	1.93 (4)	2.858 (2)	177 (3)
O1—H1*B*⋯N4^ii^	0.95 (3)	1.94 (3)	2.8905 (18)	177 (2)
N3—H3*A*⋯O1	0.93 (2)	1.82 (2)	2.7338 (17)	170 (2)
N5—H5*A*⋯N2^iii^	0.89 (2)	2.15 (2)	3.0199 (18)	167.2 (18)
O1—H1*A*⋯N7^i^	0.93 (4)	1.93 (4)	2.858 (2)	177 (3)
O1—H1*B*⋯N4^ii^	0.95 (3)	1.94 (3)	2.8905 (18)	177 (2)
N3—H3*A*⋯O1	0.93 (2)	1.82 (2)	2.7338 (17)	170 (2)
N5—H5*A*⋯N2^iii^	0.89 (2)	2.15 (2)	3.0199 (18)	167.2 (18)

Symmetry codes: (i) 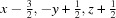; (ii) 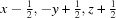; (iii) 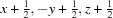.

**Table 5:** Experimental details

	(1)·2H_2_O	(1)·H_2_O
Crystal data		
Chemical formula	C_28_H_31_N_7_·2H_2_O	C_28_H_31_N_7_·H_2_O
*M*_r_	501.63	483.61
Crystal system, space group	Monoclinic, *P*2_1_/*c*	Monoclinic, *P*2_1_/*n*
Temperature (K)	130	100
*a*, *b*, *c* (Å)	8.7190 (3), 12.0891 (3), 24.6794 (7)	9.8750 (1), 22.6561 (3), 11.7917 (1)
β (°)	90.806 (3)	101.188 (1)
*V* (Å^3^)	2601.07 (13)	2588.01 (5)
*Z*	4	4
Radiation type	Cu *K*α	Cu *K*α
μ (mm^−1^)	0.67	0.63
Crystal size (mm)	0.41 × 0.19 × 0.04	0.29 × 0.16 × 0.07

Data collection		
Diffractometer	Rigaku Oxford Diffraction SuperNova, Dual, Cu at zero, Atlas	XtaLAB Synergy, Dualflex, HyPix
Absorption correction	Multi-scan (*CrysAlis PRO*; Rigaku OD, 2015 ▸)	Multi-scan (*CrysAlis PRO*; Rigaku OD, 2015 ▸)
*T*_min_, *T*_max_	0.911, 1.000	0.724, 1.000
No. of measured, independent and observed [*I* > 2σ(*I*)] reflections	15892, 5318, 4425	33621, 5468, 4622
*R*_int_	0.033	0.072
(sin θ/λ)_max_ (Å^−1^)	0.629	0.635

Refinement		
*R*[*F* ^2^ > 2σ(*F* ^2^)], *wR*(*F* ^2^), *S*	0.044, 0.119, 1.03	0.050, 0.144, 1.08
No. of reflections	5318	5468
No. of parameters	362	345
H-atom treatment	H atoms treated by a mixture of independent and constrained refinement	H atoms treated by a mixture of independent and constrained refinement
Δρ_max_, Δρ_min_ (e Å^−3^)	0.21, −0.24	0.51, −0.31

Computer programs: *CrysAlis PRO* (Rigaku OD, 2018 ▸), *SHELXT* (Sheldrick, 2015*a* ▸), *SHELXL2016/6* (Sheldrick, 2015*b* ▸), *CrystalMaker* (Palmer 2014 ▸), *Mercury*, (Macrae *et al.* 2008 ▸) and *publCIF* (Westrip, 2010 ▸).

## supplementary crystallographic information

### N,N,3-Trimethyl-4-[6-(4-methylpiperazin-1-yl)-1H,3'H-[2,5'-bibenzo[d]imidazol]-2'-yl]aniline dihydrate (1_dihydrate) Crystal data

**Table:**

C_28_H_31_N_7_·2H_2_O	*F*(000) = 1072
*M**_r_* = 501.63	*D*_x_ = 1.281 Mg m^−^^3^
Monoclinic, *P*2_1_/*c*	Cu *K*α radiation, λ = 1.54184 Å
*a* = 8.7190 (3) Å	Cell parameters from 4889 reflections
*b* = 12.0891 (3) Å	θ = 3.6–75.0°
*c* = 24.6794 (7) Å	µ = 0.67 mm^−^^1^
β = 90.806 (3)°	*T* = 130 K
*V* = 2601.07 (13) Å^3^	PLATE, brown
*Z* = 4	0.41 × 0.19 × 0.04 mm

### N,N,3-Trimethyl-4-[6-(4-methylpiperazin-1-yl)-1H,3'H-[2,5'-bibenzo[d]imidazol]-2'-yl]aniline dihydrate (1_dihydrate) Data collection

**Table:**

Rigaku Oxford Diffraction SuperNova, Dual, Cu at zero, Atlas diffractometer	5318 independent reflections
Radiation source: micro-focus sealed X-ray tube, SuperNova (Cu) X-ray Source	4425 reflections with *I* > 2σ(*I*)
Mirror monochromator	*R*_int_ = 0.033
Detector resolution: 10.2273 pixels mm^-1^	θ_max_ = 75.8°, θ_min_ = 3.6°
ω scans	*h* = −10→10
Absorption correction: multi-scan (CrysAlisPro; Rigaku OD, 2015)	*k* = −15→14
*T*_min_ = 0.911, *T*_max_ = 1.000	*l* = −16→30
15892 measured reflections	

### N,N,3-Trimethyl-4-[6-(4-methylpiperazin-1-yl)-1H,3'H-[2,5'-bibenzo[d]imidazol]-2'-yl]aniline dihydrate (1_dihydrate) Refinement

**Table:**

Refinement on *F*^2^	0 restraints
Least-squares matrix: full	Hydrogen site location: mixed
*R*[*F*^2^ > 2σ(*F*^2^)] = 0.044	H atoms treated by a mixture of independent and constrained refinement
*wR*(*F*^2^) = 0.119	*w* = 1/[σ^2^(*F*_o_^2^) + (0.0583*P*)^2^ + 0.7255*P*] where *P* = (*F*_o_^2^ + 2*F*_c_^2^)/3
*S* = 1.03	(Δ/σ)_max_ = 0.001
5318 reflections	Δρ_max_ = 0.21 e Å^−^^3^
362 parameters	Δρ_min_ = −0.24 e Å^−^^3^

### N,N,3-Trimethyl-4-[6-(4-methylpiperazin-1-yl)-1H,3'H-[2,5'-bibenzo[d]imidazol]-2'-yl]aniline dihydrate (1_dihydrate) Special details

**Table:**

Geometry. All esds (except the esd in the dihedral angle between two l.s. planes) are estimated using the full covariance matrix. The cell esds are taken into account individually in the estimation of esds in distances, angles and torsion angles; correlations between esds in cell parameters are only used when they are defined by crystal symmetry. An approximate (isotropic) treatment of cell esds is used for estimating esds involving l.s. planes.

### N,N,3-Trimethyl-4-[6-(4-methylpiperazin-1-yl)-1H,3'H-[2,5'-bibenzo[d]imidazol]-2'-yl]aniline dihydrate (1_dihydrate) Fractional atomic coordinates and isotropic or equivalent isotropic displacement parameters (Å^2^)

**Table:**

	*x*	*y*	*z*	*U*_iso_*/*U*_eq_	
C1	0.69302 (17)	0.17764 (11)	0.65874 (5)	0.0243 (3)	
C2	0.79427 (17)	0.11850 (12)	0.69258 (6)	0.0258 (3)	
C3	0.81413 (17)	0.00556 (12)	0.68405 (6)	0.0277 (3)	
H3	0.881999	−0.033944	0.707464	0.033*	
C4	0.73832 (17)	−0.05232 (12)	0.64241 (6)	0.0270 (3)	
C5	0.64357 (18)	0.00932 (12)	0.60713 (6)	0.0287 (3)	
H5	0.594461	−0.026113	0.577227	0.034*	
C6	0.62141 (17)	0.12092 (12)	0.61563 (6)	0.0268 (3)	
H6	0.555661	0.160674	0.591572	0.032*	
C7	0.65276 (17)	0.29433 (11)	0.66734 (5)	0.0242 (3)	
C8	0.56121 (17)	0.46080 (11)	0.64655 (5)	0.0244 (3)	
C9	0.49737 (18)	0.55453 (11)	0.62294 (5)	0.0257 (3)	
H9	0.479766	0.559215	0.584935	0.031*	
C10	0.46019 (17)	0.64149 (11)	0.65739 (6)	0.0253 (3)	
C11	0.48861 (18)	0.63285 (12)	0.71361 (6)	0.0281 (3)	
H11	0.464455	0.693767	0.736265	0.034*	
C12	0.55047 (19)	0.53861 (12)	0.73678 (5)	0.0287 (3)	
H12	0.566304	0.533360	0.774861	0.034*	
C13	0.58903 (17)	0.45133 (11)	0.70256 (5)	0.0249 (3)	
C14	0.38673 (17)	0.74118 (11)	0.63489 (5)	0.0248 (3)	
C15	0.27354 (17)	0.90514 (12)	0.63245 (6)	0.0253 (3)	
C16	0.21044 (18)	1.00914 (12)	0.64186 (6)	0.0272 (3)	
H16	0.196434	1.035778	0.677671	0.033*	
C17	0.16846 (17)	1.07293 (12)	0.59683 (6)	0.0287 (3)	
C18	0.1847 (2)	1.02899 (13)	0.54424 (6)	0.0330 (3)	
H18	0.153195	1.072271	0.513946	0.040*	
C19	0.2453 (2)	0.92474 (13)	0.53543 (6)	0.0318 (3)	
H19	0.254261	0.896344	0.499724	0.038*	
C20	0.29259 (18)	0.86253 (12)	0.58001 (6)	0.0266 (3)	
C21	0.2269 (2)	1.25553 (12)	0.63172 (7)	0.0340 (3)	
H21A	0.314059	1.266312	0.607004	0.041*	
H21B	0.266620	1.219978	0.665212	0.041*	
C22	0.1582 (2)	1.36723 (13)	0.64567 (7)	0.0378 (4)	
H22A	0.075915	1.357214	0.672463	0.045*	
H22B	0.238348	1.415039	0.662181	0.045*	
C23	−0.0209 (2)	1.34796 (14)	0.57307 (9)	0.0430 (4)	
H23A	−0.065811	1.383431	0.540348	0.052*	
H23B	−0.104284	1.336053	0.599265	0.052*	
C24	0.0488 (2)	1.23730 (13)	0.55780 (8)	0.0390 (4)	
H24A	−0.030972	1.189565	0.541029	0.047*	
H24B	0.130560	1.248921	0.530945	0.047*	
C25	0.88799 (19)	0.17115 (13)	0.73735 (6)	0.0322 (3)	
H25A	0.832606	0.165570	0.771519	0.048*	
H25B	0.905626	0.249222	0.728749	0.048*	
H25C	0.986732	0.132916	0.740906	0.048*	
C26	0.7615 (2)	−0.21319 (13)	0.58283 (7)	0.0370 (4)	
H26A	0.686373	−0.176899	0.558870	0.056*	
H26B	0.739873	−0.292682	0.584254	0.056*	
H26C	0.864900	−0.201421	0.568883	0.056*	
C27	0.8446 (2)	−0.22581 (13)	0.67654 (7)	0.0398 (4)	
H27A	0.953129	−0.208560	0.671010	0.060*	
H27B	0.828282	−0.305536	0.672196	0.060*	
H27C	0.815422	−0.203391	0.713128	0.060*	
C28	0.0293 (2)	1.52855 (14)	0.61022 (9)	0.0481 (5)	
H28A	0.109769	1.577017	0.624928	0.072*	
H28B	−0.051069	1.519072	0.637206	0.072*	
H28C	−0.015043	1.561809	0.577347	0.072*	
N1	0.75161 (17)	−0.16655 (10)	0.63700 (5)	0.0326 (3)	
N2	0.60315 (15)	0.35974 (10)	0.62531 (5)	0.0247 (3)	
N3	0.64741 (15)	0.34701 (10)	0.71470 (5)	0.0258 (3)	
N4	0.33347 (15)	0.82541 (9)	0.66670 (5)	0.0252 (3)	
N5	0.36288 (15)	0.75965 (10)	0.58267 (5)	0.0276 (3)	
N6	0.11305 (15)	1.18284 (10)	0.60580 (5)	0.0304 (3)	
N7	0.09523 (16)	1.42065 (11)	0.59706 (6)	0.0340 (3)	
O1	0.71569 (15)	0.55306 (10)	0.49523 (5)	0.0367 (3)	
O2	0.43370 (15)	0.65458 (9)	0.48653 (4)	0.0320 (2)	
H1A	0.685 (3)	0.481 (2)	0.4987 (10)	0.064 (7)*	
H1B	0.768 (4)	0.559 (3)	0.4623 (13)	0.088 (10)*	
H2	0.5989 (19)	0.3436 (14)	0.5915 (7)	0.024 (4)*	
H2A	0.413 (3)	0.691 (2)	0.5155 (9)	0.049 (6)*	
H2B	0.532 (3)	0.631 (2)	0.4886 (11)	0.079 (9)*	
H4A	0.339 (2)	0.8289 (15)	0.7019 (8)	0.029 (4)*	

### N,N,3-Trimethyl-4-[6-(4-methylpiperazin-1-yl)-1H,3'H-[2,5'-bibenzo[d]imidazol]-2'-yl]aniline dihydrate (1_dihydrate) Atomic displacement parameters (Å^2^)

**Table:**

	*U*^11^	*U*^22^	*U*^33^	*U*^12^	*U*^13^	*U*^23^
C1	0.0348 (7)	0.0171 (6)	0.0212 (6)	0.0013 (5)	0.0027 (5)	0.0007 (5)
C2	0.0335 (7)	0.0217 (7)	0.0222 (6)	−0.0003 (6)	0.0013 (5)	0.0005 (5)
C3	0.0346 (7)	0.0222 (7)	0.0262 (7)	0.0041 (6)	−0.0018 (6)	0.0027 (5)
C4	0.0350 (7)	0.0191 (7)	0.0269 (7)	0.0020 (5)	0.0028 (6)	0.0002 (5)
C5	0.0392 (8)	0.0214 (7)	0.0256 (7)	0.0006 (6)	−0.0029 (6)	−0.0029 (5)
C6	0.0367 (7)	0.0211 (7)	0.0226 (6)	0.0037 (6)	−0.0010 (5)	0.0005 (5)
C7	0.0345 (7)	0.0182 (6)	0.0199 (6)	0.0004 (5)	0.0013 (5)	0.0017 (5)
C8	0.0369 (7)	0.0177 (6)	0.0186 (6)	−0.0003 (5)	0.0027 (5)	−0.0012 (5)
C9	0.0418 (8)	0.0188 (6)	0.0163 (6)	0.0023 (6)	0.0001 (5)	0.0002 (5)
C10	0.0382 (7)	0.0169 (6)	0.0207 (6)	0.0011 (5)	−0.0001 (5)	−0.0002 (5)
C11	0.0459 (8)	0.0182 (6)	0.0201 (7)	0.0032 (6)	0.0001 (6)	−0.0030 (5)
C12	0.0477 (8)	0.0227 (7)	0.0156 (6)	0.0038 (6)	0.0000 (6)	−0.0002 (5)
C13	0.0390 (7)	0.0171 (6)	0.0186 (6)	0.0010 (5)	0.0003 (5)	0.0018 (5)
C14	0.0389 (7)	0.0174 (6)	0.0181 (6)	0.0006 (5)	0.0004 (5)	−0.0016 (5)
C15	0.0350 (7)	0.0190 (6)	0.0219 (7)	0.0001 (5)	−0.0005 (5)	0.0015 (5)
C16	0.0379 (7)	0.0194 (6)	0.0243 (7)	0.0014 (6)	0.0019 (5)	−0.0011 (5)
C17	0.0333 (7)	0.0193 (7)	0.0335 (8)	0.0008 (6)	−0.0003 (6)	0.0014 (6)
C18	0.0475 (9)	0.0232 (7)	0.0282 (7)	0.0007 (6)	−0.0072 (6)	0.0053 (6)
C19	0.0495 (9)	0.0242 (7)	0.0216 (7)	0.0020 (6)	−0.0041 (6)	−0.0009 (5)
C20	0.0392 (8)	0.0189 (6)	0.0217 (7)	0.0002 (6)	−0.0011 (6)	−0.0002 (5)
C21	0.0434 (9)	0.0213 (7)	0.0369 (8)	0.0041 (6)	−0.0054 (7)	−0.0022 (6)
C22	0.0536 (10)	0.0219 (7)	0.0381 (9)	0.0051 (7)	0.0041 (7)	−0.0010 (6)
C23	0.0373 (8)	0.0280 (8)	0.0634 (12)	0.0063 (7)	−0.0037 (8)	0.0076 (8)
C24	0.0429 (9)	0.0237 (7)	0.0498 (10)	0.0025 (7)	−0.0131 (7)	0.0034 (7)
C25	0.0402 (8)	0.0264 (7)	0.0297 (8)	0.0028 (6)	−0.0055 (6)	−0.0016 (6)
C26	0.0538 (10)	0.0215 (7)	0.0358 (8)	0.0046 (7)	0.0007 (7)	−0.0045 (6)
C27	0.0573 (10)	0.0217 (7)	0.0403 (9)	0.0090 (7)	−0.0062 (8)	0.0018 (6)
C28	0.0591 (11)	0.0256 (8)	0.0598 (12)	0.0146 (8)	0.0114 (9)	0.0020 (8)
N1	0.0474 (8)	0.0185 (6)	0.0318 (7)	0.0049 (5)	−0.0021 (6)	−0.0010 (5)
N2	0.0410 (7)	0.0173 (5)	0.0158 (6)	0.0035 (5)	0.0012 (5)	−0.0006 (4)
N3	0.0420 (7)	0.0171 (5)	0.0184 (5)	0.0017 (5)	0.0010 (5)	0.0012 (4)
N4	0.0411 (7)	0.0170 (5)	0.0175 (6)	0.0026 (5)	0.0007 (5)	−0.0003 (4)
N5	0.0446 (7)	0.0185 (6)	0.0198 (6)	0.0031 (5)	−0.0013 (5)	−0.0006 (4)
N6	0.0355 (6)	0.0193 (6)	0.0362 (7)	0.0027 (5)	−0.0012 (5)	0.0027 (5)
N7	0.0397 (7)	0.0197 (6)	0.0429 (8)	0.0059 (5)	0.0069 (6)	0.0029 (5)
O1	0.0456 (6)	0.0286 (6)	0.0360 (6)	0.0002 (5)	0.0037 (5)	−0.0011 (5)
O2	0.0499 (7)	0.0276 (5)	0.0186 (5)	0.0081 (5)	−0.0014 (4)	−0.0036 (4)

### N,N,3-Trimethyl-4-[6-(4-methylpiperazin-1-yl)-1H,3'H-[2,5'-bibenzo[d]imidazol]-2'-yl]aniline dihydrate (1_dihydrate) Geometric parameters (Å, º)

**Table:**

C1—C2	1.403 (2)	C19—H19	0.9500
C1—C6	1.405 (2)	C20—N5	1.3877 (19)
C1—C7	1.4698 (18)	C21—N6	1.466 (2)
C2—C3	1.393 (2)	C21—C22	1.519 (2)
C2—C25	1.506 (2)	C21—H21A	0.9900
C3—C4	1.401 (2)	C21—H21B	0.9900
C3—H3	0.9500	C22—N7	1.463 (2)
C4—N1	1.3923 (18)	C22—H22A	0.9900
C4—C5	1.406 (2)	C22—H22B	0.9900
C5—C6	1.379 (2)	C23—N7	1.460 (2)
C5—H5	0.9500	C23—C24	1.519 (2)
C6—H6	0.9500	C23—H23A	0.9900
C7—N2	1.3695 (17)	C23—H23B	0.9900
C7—N3	1.3325 (18)	C24—N6	1.460 (2)
C8—N2	1.3808 (17)	C24—H24A	0.9900
C8—C9	1.3871 (19)	C24—H24B	0.9900
C8—C13	1.4047 (19)	C25—H25A	0.9800
C9—C10	1.3931 (19)	C25—H25B	0.9800
C9—H9	0.9500	C25—H25C	0.9800
C10—C11	1.410 (2)	C26—N1	1.455 (2)
C10—C14	1.4701 (19)	C26—H26A	0.9800
C11—C12	1.381 (2)	C26—H26B	0.9800
C11—H11	0.9500	C26—H26C	0.9800
C12—C13	1.3957 (19)	C27—N1	1.449 (2)
C12—H12	0.9500	C27—H27A	0.9800
C13—N3	1.3910 (18)	C27—H27B	0.9800
C14—N4	1.3708 (18)	C27—H27C	0.9800
C14—N5	1.3215 (18)	C28—N7	1.464 (2)
C15—N4	1.3799 (18)	C28—H28A	0.9800
C15—C16	1.393 (2)	C28—H28B	0.9800
C15—C20	1.4051 (19)	C28—H28C	0.9800
C16—C17	1.397 (2)	N2—H2	0.856 (18)
C16—H16	0.9500	N4—H4A	0.870 (19)
C17—C18	1.412 (2)	O1—H1A	0.91 (3)
C17—N6	1.4321 (19)	O1—H1B	0.94 (3)
C18—C19	1.385 (2)	O2—H2A	0.86 (2)
C18—H18	0.9500	O2—H2B	0.90 (3)
C19—C20	1.390 (2)		
			
C2—C1—C6	118.14 (12)	C22—C21—H21B	109.3
C2—C1—C7	123.56 (13)	H21A—C21—H21B	108.0
C6—C1—C7	118.25 (13)	N7—C22—C21	110.56 (14)
C3—C2—C1	119.22 (13)	N7—C22—H22A	109.5
C3—C2—C25	117.25 (13)	C21—C22—H22A	109.5
C1—C2—C25	123.50 (13)	N7—C22—H22B	109.5
C2—C3—C4	122.83 (13)	C21—C22—H22B	109.5
C2—C3—H3	118.6	H22A—C22—H22B	108.1
C4—C3—H3	118.6	N7—C23—C24	110.66 (14)
N1—C4—C3	121.77 (13)	N7—C23—H23A	109.5
N1—C4—C5	121.03 (13)	C24—C23—H23A	109.5
C3—C4—C5	117.17 (13)	N7—C23—H23B	109.5
C6—C5—C4	120.44 (13)	C24—C23—H23B	109.5
C6—C5—H5	119.8	H23A—C23—H23B	108.1
C4—C5—H5	119.8	N6—C24—C23	110.25 (15)
C5—C6—C1	122.07 (13)	N6—C24—H24A	109.6
C5—C6—H6	119.0	C23—C24—H24A	109.6
C1—C6—H6	119.0	N6—C24—H24B	109.6
N3—C7—N2	111.95 (12)	C23—C24—H24B	109.6
N3—C7—C1	126.63 (12)	H24A—C24—H24B	108.1
N2—C7—C1	121.22 (12)	C2—C25—H25A	109.5
N2—C8—C9	132.08 (13)	C2—C25—H25B	109.5
N2—C8—C13	104.98 (12)	H25A—C25—H25B	109.5
C9—C8—C13	122.86 (12)	C2—C25—H25C	109.5
C8—C9—C10	117.10 (12)	H25A—C25—H25C	109.5
C8—C9—H9	121.4	H25B—C25—H25C	109.5
C10—C9—H9	121.4	N1—C26—H26A	109.5
C9—C10—C11	120.36 (13)	N1—C26—H26B	109.5
C9—C10—C14	119.47 (12)	H26A—C26—H26B	109.5
C11—C10—C14	120.14 (12)	N1—C26—H26C	109.5
C12—C11—C10	122.06 (13)	H26A—C26—H26C	109.5
C12—C11—H11	119.0	H26B—C26—H26C	109.5
C10—C11—H11	119.0	N1—C27—H27A	109.5
C11—C12—C13	117.98 (13)	N1—C27—H27B	109.5
C11—C12—H12	121.0	H27A—C27—H27B	109.5
C13—C12—H12	121.0	N1—C27—H27C	109.5
N3—C13—C12	130.21 (13)	H27A—C27—H27C	109.5
N3—C13—C8	110.09 (12)	H27B—C27—H27C	109.5
C12—C13—C8	119.61 (13)	N7—C28—H28A	109.5
N5—C14—N4	112.53 (12)	N7—C28—H28B	109.5
N5—C14—C10	124.66 (13)	H28A—C28—H28B	109.5
N4—C14—C10	122.81 (12)	N7—C28—H28C	109.5
N4—C15—C16	132.48 (13)	H28A—C28—H28C	109.5
N4—C15—C20	105.04 (12)	H28B—C28—H28C	109.5
C16—C15—C20	122.44 (13)	C4—N1—C27	118.18 (13)
C15—C16—C17	117.71 (13)	C4—N1—C26	118.60 (13)
C15—C16—H16	121.1	C27—N1—C26	112.78 (13)
C17—C16—H16	121.1	C7—N2—C8	107.81 (11)
C16—C17—C18	119.68 (13)	C7—N2—H2	127.9 (12)
C16—C17—N6	118.32 (13)	C8—N2—H2	124.2 (12)
C18—C17—N6	121.99 (13)	C7—N3—C13	105.17 (11)
C19—C18—C17	122.03 (14)	C14—N4—C15	107.25 (12)
C19—C18—H18	119.0	C14—N4—H4A	126.4 (12)
C17—C18—H18	119.0	C15—N4—H4A	126.3 (12)
C18—C19—C20	118.53 (14)	C14—N5—C20	105.13 (12)
C18—C19—H19	120.7	C17—N6—C24	114.80 (13)
C20—C19—H19	120.7	C17—N6—C21	113.32 (12)
N5—C20—C19	130.40 (13)	C24—N6—C21	109.48 (12)
N5—C20—C15	110.05 (12)	C23—N7—C22	108.50 (13)
C19—C20—C15	119.54 (13)	C23—N7—C28	110.72 (14)
N6—C21—C22	111.44 (14)	C22—N7—C28	110.83 (14)
N6—C21—H21A	109.3	H1A—O1—H1B	108 (2)
C22—C21—H21A	109.3	H2A—O2—H2B	109 (2)
N6—C21—H21B	109.3		
			
C6—C1—C2—C3	−3.2 (2)	C18—C19—C20—N5	−176.53 (16)
C7—C1—C2—C3	174.06 (13)	C18—C19—C20—C15	2.0 (2)
C6—C1—C2—C25	174.91 (14)	N4—C15—C20—N5	−0.20 (17)
C7—C1—C2—C25	−7.8 (2)	C16—C15—C20—N5	177.83 (14)
C1—C2—C3—C4	0.8 (2)	N4—C15—C20—C19	−178.99 (14)
C25—C2—C3—C4	−177.47 (14)	C16—C15—C20—C19	−1.0 (2)
C2—C3—C4—N1	−175.39 (14)	N6—C21—C22—N7	−57.69 (19)
C2—C3—C4—C5	2.5 (2)	N7—C23—C24—N6	60.2 (2)
N1—C4—C5—C6	174.59 (14)	C3—C4—N1—C27	1.2 (2)
C3—C4—C5—C6	−3.3 (2)	C5—C4—N1—C27	−176.68 (15)
C4—C5—C6—C1	0.9 (2)	C3—C4—N1—C26	−141.25 (15)
C2—C1—C6—C5	2.4 (2)	C5—C4—N1—C26	40.9 (2)
C7—C1—C6—C5	−175.00 (14)	N3—C7—N2—C8	−0.72 (17)
C2—C1—C7—N3	−30.0 (2)	C1—C7—N2—C8	174.58 (13)
C6—C1—C7—N3	147.26 (15)	C9—C8—N2—C7	−176.60 (16)
C2—C1—C7—N2	155.44 (14)	C13—C8—N2—C7	0.18 (16)
C6—C1—C7—N2	−27.3 (2)	N2—C7—N3—C13	0.92 (17)
N2—C8—C9—C10	176.15 (15)	C1—C7—N3—C13	−174.06 (14)
C13—C8—C9—C10	−0.1 (2)	C12—C13—N3—C7	175.75 (16)
C8—C9—C10—C11	0.5 (2)	C8—C13—N3—C7	−0.79 (17)
C8—C9—C10—C14	−177.65 (13)	N5—C14—N4—C15	−1.25 (18)
C9—C10—C11—C12	−1.4 (2)	C10—C14—N4—C15	179.45 (13)
C14—C10—C11—C12	176.79 (15)	C16—C15—N4—C14	−176.92 (16)
C10—C11—C12—C13	1.7 (2)	C20—C15—N4—C14	0.83 (16)
C11—C12—C13—N3	−177.60 (15)	N4—C14—N5—C20	1.09 (17)
C11—C12—C13—C8	−1.3 (2)	C10—C14—N5—C20	−179.62 (14)
N2—C8—C13—N3	0.38 (17)	C19—C20—N5—C14	178.09 (17)
C9—C8—C13—N3	177.53 (14)	C15—C20—N5—C14	−0.53 (17)
N2—C8—C13—C12	−176.59 (14)	C16—C17—N6—C24	−170.61 (14)
C9—C8—C13—C12	0.6 (2)	C18—C17—N6—C24	10.8 (2)
C9—C10—C14—N5	−5.7 (2)	C16—C17—N6—C21	62.58 (19)
C11—C10—C14—N5	176.15 (15)	C18—C17—N6—C21	−115.97 (17)
C9—C10—C14—N4	173.54 (14)	C23—C24—N6—C17	174.17 (14)
C11—C10—C14—N4	−4.6 (2)	C23—C24—N6—C21	−57.08 (18)
N4—C15—C16—C17	176.01 (15)	C22—C21—N6—C17	−174.13 (13)
C20—C15—C16—C17	−1.4 (2)	C22—C21—N6—C24	56.31 (18)
C15—C16—C17—C18	2.7 (2)	C24—C23—N7—C22	−59.96 (19)
C15—C16—C17—N6	−175.89 (13)	C24—C23—N7—C28	178.21 (15)
C16—C17—C18—C19	−1.7 (2)	C21—C22—N7—C23	58.40 (18)
N6—C17—C18—C19	176.81 (15)	C21—C22—N7—C28	−179.83 (15)
C17—C18—C19—C20	−0.7 (3)		

### N,N,3-Trimethyl-4-[6-(4-methylpiperazin-1-yl)-1H,3'H-[2,5'-bibenzo[d]imidazol]-2'-yl]aniline dihydrate (1_dihydrate) Hydrogen-bond geometry (Å, º)

**Table:**

*D*—H···*A*	*D*—H	H···*A*	*D*···*A*	*D*—H···*A*
C23—H23*B*···N2^i^	0.99	2.65	3.542 (2)	149
O1—H1*A*···O2^ii^	0.91 (3)	1.98 (3)	2.8665 (17)	164 (2)
O1—H1*B*···N7^iii^	0.94 (3)	1.91 (3)	2.8482 (18)	170 (3)
N2—H2···O2^ii^	0.856 (18)	1.944 (18)	2.7797 (15)	165.0 (17)
O2—H2*A*···N5	0.86 (2)	1.91 (2)	2.7685 (16)	175 (2)
O2—H2*B*···O1	0.90 (3)	1.86 (3)	2.7537 (18)	168 (3)
N4—H4*A*···N3^iv^	0.870 (19)	2.072 (19)	2.9411 (16)	176.7 (17)

Symmetry codes: (i) *x*−1, *y*+1, *z*; (ii) −*x*+1, −*y*+1, −*z*+1; (iii) −*x*+1, −*y*+2, −*z*+1; (iv) −*x*+1, *y*+1/2, −*z*+3/2.

### N,N,3-Trimethyl-4-[6-(4-methylpiperazin-1-yl)-1H,3'H-[2,5'-bibenzo[d]imidazol]-2'-yl]aniline monohydrate (1_hydrate) Crystal data

**Table:**

C_28_H_31_N_7_·H_2_O	*F*(000) = 1032
*M**_r_* = 483.61	*D*_x_ = 1.241 Mg m^−^^3^
Monoclinic, *P*2_1_/*n*	Cu *K*α radiation, λ = 1.54184 Å
*a* = 9.8750 (1) Å	Cell parameters from 12480 reflections
*b* = 22.6561 (3) Å	θ = 4.3–77.4°
*c* = 11.7917 (1) Å	µ = 0.63 mm^−^^1^
β = 101.188 (1)°	*T* = 100 K
*V* = 2588.01 (5) Å^3^	ROD, brown
*Z* = 4	0.29 × 0.16 × 0.07 mm

### N,N,3-Trimethyl-4-[6-(4-methylpiperazin-1-yl)-1H,3'H-[2,5'-bibenzo[d]imidazol]-2'-yl]aniline monohydrate (1_hydrate) Data collection

**Table:**

XtaLAB Synergy, Dualflex, HyPix diffractometer	5468 independent reflections
Radiation source: micro-focus sealed X-ray tube, PhotonJet (Cu) X-ray Source	4622 reflections with *I* > 2σ(*I*)
Mirror monochromator	*R*_int_ = 0.072
ω scans	θ_max_ = 78.2°, θ_min_ = 3.9°
Absorption correction: multi-scan (CrysAlisPro; Rigaku OD, 2015)	*h* = −12→12
*T*_min_ = 0.724, *T*_max_ = 1.000	*k* = −13→28
33621 measured reflections	*l* = −14→14

### N,N,3-Trimethyl-4-[6-(4-methylpiperazin-1-yl)-1H,3'H-[2,5'-bibenzo[d]imidazol]-2'-yl]aniline monohydrate (1_hydrate) Refinement

**Table:**

Refinement on *F*^2^	0 restraints
Least-squares matrix: full	Hydrogen site location: mixed
*R*[*F*^2^ > 2σ(*F*^2^)] = 0.050	H atoms treated by a mixture of independent and constrained refinement
*wR*(*F*^2^) = 0.144	*w* = 1/[σ^2^(*F*_o_^2^) + (0.0744*P*)^2^ + 0.8256*P*] where *P* = (*F*_o_^2^ + 2*F*_c_^2^)/3
*S* = 1.08	(Δ/σ)_max_ < 0.001
5468 reflections	Δρ_max_ = 0.51 e Å^−^^3^
345 parameters	Δρ_min_ = −0.31 e Å^−^^3^

### N,N,3-Trimethyl-4-[6-(4-methylpiperazin-1-yl)-1H,3'H-[2,5'-bibenzo[d]imidazol]-2'-yl]aniline monohydrate (1_hydrate) Special details

**Table:**

Geometry. All esds (except the esd in the dihedral angle between two l.s. planes) are estimated using the full covariance matrix. The cell esds are taken into account individually in the estimation of esds in distances, angles and torsion angles; correlations between esds in cell parameters are only used when they are defined by crystal symmetry. An approximate (isotropic) treatment of cell esds is used for estimating esds involving l.s. planes.

### N,N,3-Trimethyl-4-[6-(4-methylpiperazin-1-yl)-1H,3'H-[2,5'-bibenzo[d]imidazol]-2'-yl]aniline monohydrate (1_hydrate) Fractional atomic coordinates and isotropic or equivalent isotropic displacement parameters (Å^2^)

**Table:**

	*x*	*y*	*z*	*U*_iso_*/*U*_eq_	
C1	0.01456 (15)	0.36648 (7)	0.26721 (12)	0.0241 (3)	
C2	0.00764 (15)	0.41121 (7)	0.18284 (13)	0.0248 (3)	
C3	−0.11128 (16)	0.44501 (7)	0.15471 (13)	0.0271 (3)	
H3	−0.114786	0.474253	0.098861	0.032*	
C4	−0.22698 (16)	0.43717 (7)	0.20671 (13)	0.0281 (3)	
C5	−0.22147 (16)	0.39030 (8)	0.28626 (14)	0.0301 (3)	
H5	−0.297515	0.382171	0.319436	0.036*	
C6	−0.10332 (16)	0.35642 (7)	0.31502 (13)	0.0273 (3)	
H6	−0.101718	0.325855	0.367918	0.033*	
C7	0.14024 (15)	0.33245 (7)	0.31155 (12)	0.0229 (3)	
C8	0.34140 (15)	0.29191 (7)	0.33857 (12)	0.0228 (3)	
C9	0.47236 (15)	0.27141 (7)	0.33039 (13)	0.0241 (3)	
H9	0.508496	0.277953	0.264282	0.029*	
C10	0.54734 (15)	0.24077 (7)	0.42464 (12)	0.0234 (3)	
C11	0.49178 (15)	0.23108 (7)	0.52482 (12)	0.0236 (3)	
H11	0.544475	0.210978	0.586806	0.028*	
C12	0.36138 (15)	0.25057 (7)	0.53357 (12)	0.0241 (3)	
H12	0.324416	0.243352	0.599015	0.029*	
C13	0.28837 (15)	0.28160 (6)	0.43910 (13)	0.0228 (3)	
C14	0.68280 (15)	0.21645 (6)	0.41738 (12)	0.0223 (3)	
C15	0.85786 (15)	0.18224 (6)	0.35357 (13)	0.0228 (3)	
C16	0.95061 (15)	0.16217 (7)	0.28615 (13)	0.0252 (3)	
H16	0.929305	0.164904	0.205961	0.030*	
C17	1.07574 (15)	0.13799 (6)	0.34208 (13)	0.0244 (3)	
C18	1.10692 (15)	0.13588 (7)	0.46444 (13)	0.0263 (3)	
H18	1.191352	0.120358	0.500963	0.032*	
C19	1.01680 (16)	0.15595 (7)	0.53104 (13)	0.0266 (3)	
H19	1.039331	0.154514	0.611317	0.032*	
C20	0.89077 (15)	0.17849 (6)	0.47443 (13)	0.0231 (3)	
C21	1.13723 (19)	0.11372 (9)	0.15611 (15)	0.0368 (4)	
H21A	1.145758	0.153897	0.129953	0.044*	
H21B	1.042476	0.101140	0.129598	0.044*	
C22	1.2334 (2)	0.07369 (9)	0.10524 (16)	0.0392 (4)	
H22A	1.219614	0.033188	0.127120	0.047*	
H22B	1.210701	0.076123	0.021566	0.047*	
C23	1.41019 (19)	0.08795 (8)	0.27129 (15)	0.0361 (4)	
H23A	1.505209	0.100072	0.298135	0.043*	
H23B	1.400472	0.047877	0.297564	0.043*	
C24	1.31556 (17)	0.12828 (8)	0.32169 (15)	0.0329 (4)	
H24A	1.337798	0.126002	0.405395	0.039*	
H24B	1.329549	0.168699	0.299402	0.039*	
C25	0.12476 (17)	0.42521 (7)	0.12195 (15)	0.0314 (3)	
H25A	0.134451	0.393832	0.069391	0.047*	
H25B	0.208865	0.429238	0.177969	0.047*	
H25C	0.105481	0.461455	0.079748	0.047*	
C26	−0.3390 (2)	0.52112 (8)	0.09748 (18)	0.0413 (4)	
H26A	−0.265556	0.547916	0.127881	0.062*	
H26B	−0.425557	0.541683	0.085972	0.062*	
H26C	−0.324627	0.505577	0.024996	0.062*	
C27	−0.45043 (19)	0.47056 (10)	0.24402 (18)	0.0435 (5)	
H27A	−0.493893	0.432503	0.234141	0.065*	
H27B	−0.517460	0.500604	0.216914	0.065*	
H27C	−0.412649	0.476904	0.324428	0.065*	
C28	1.4660 (2)	0.04941 (9)	0.09566 (18)	0.0452 (5)	
H28A	1.452215	0.009836	0.120255	0.068*	
H28B	1.560756	0.060461	0.121696	0.068*	
H28C	1.443296	0.051435	0.012819	0.068*	
N1	−0.34070 (14)	0.47306 (7)	0.17847 (13)	0.0347 (3)	
N2	0.24614 (13)	0.32318 (6)	0.25861 (11)	0.0239 (3)	
N3	0.16059 (13)	0.30763 (6)	0.41935 (11)	0.0236 (3)	
N4	0.72636 (13)	0.20628 (6)	0.31942 (11)	0.0244 (3)	
N5	0.77794 (13)	0.20066 (6)	0.51316 (11)	0.0231 (3)	
N6	1.17078 (13)	0.11179 (6)	0.28103 (11)	0.0268 (3)	
N7	1.37733 (15)	0.08964 (6)	0.14459 (12)	0.0324 (3)	
O1	0.03124 (13)	0.30826 (6)	0.60438 (10)	0.0343 (3)	
H1A	−0.016 (4)	0.3420 (17)	0.618 (3)	0.091 (11)*	
H1B	0.097 (3)	0.3046 (11)	0.675 (2)	0.051 (7)*	
H3A	0.107 (2)	0.3088 (9)	0.476 (2)	0.039 (6)*	
H5A	0.765 (2)	0.1996 (9)	0.585 (2)	0.031 (5)*	

### N,N,3-Trimethyl-4-[6-(4-methylpiperazin-1-yl)-1H,3'H-[2,5'-bibenzo[d]imidazol]-2'-yl]aniline monohydrate (1_hydrate) Atomic displacement parameters (Å^2^)

**Table:**

	*U*^11^	*U*^22^	*U*^33^	*U*^12^	*U*^13^	*U*^23^
C1	0.0212 (7)	0.0309 (7)	0.0192 (7)	0.0020 (6)	0.0016 (5)	−0.0043 (5)
C2	0.0229 (7)	0.0282 (7)	0.0219 (7)	0.0014 (6)	0.0013 (5)	−0.0035 (5)
C3	0.0256 (7)	0.0285 (7)	0.0254 (7)	0.0015 (6)	0.0006 (6)	−0.0019 (6)
C4	0.0221 (7)	0.0341 (8)	0.0262 (7)	0.0037 (6)	−0.0001 (6)	−0.0059 (6)
C5	0.0221 (7)	0.0427 (9)	0.0251 (7)	0.0027 (6)	0.0041 (6)	−0.0043 (6)
C6	0.0225 (7)	0.0362 (8)	0.0225 (7)	0.0016 (6)	0.0029 (6)	0.0003 (6)
C7	0.0207 (7)	0.0295 (7)	0.0183 (6)	0.0013 (5)	0.0034 (5)	−0.0011 (5)
C8	0.0207 (7)	0.0302 (7)	0.0172 (6)	0.0014 (5)	0.0025 (5)	0.0001 (5)
C9	0.0215 (7)	0.0324 (7)	0.0188 (7)	0.0011 (6)	0.0053 (5)	0.0016 (6)
C10	0.0205 (7)	0.0285 (7)	0.0212 (7)	−0.0004 (5)	0.0043 (5)	0.0002 (5)
C11	0.0226 (7)	0.0293 (7)	0.0182 (7)	0.0014 (6)	0.0018 (5)	0.0016 (5)
C12	0.0239 (7)	0.0307 (7)	0.0183 (7)	0.0001 (6)	0.0058 (5)	−0.0003 (5)
C13	0.0195 (7)	0.0292 (7)	0.0200 (7)	0.0011 (5)	0.0042 (5)	−0.0009 (5)
C14	0.0200 (7)	0.0274 (7)	0.0191 (7)	0.0005 (5)	0.0028 (5)	0.0011 (5)
C15	0.0201 (7)	0.0271 (7)	0.0212 (7)	−0.0002 (5)	0.0036 (5)	0.0020 (5)
C16	0.0242 (7)	0.0331 (8)	0.0187 (7)	0.0017 (6)	0.0054 (5)	0.0017 (6)
C17	0.0226 (7)	0.0272 (7)	0.0242 (7)	−0.0002 (5)	0.0068 (6)	0.0002 (5)
C18	0.0210 (7)	0.0325 (8)	0.0244 (7)	0.0025 (6)	0.0017 (6)	−0.0007 (6)
C19	0.0238 (7)	0.0348 (8)	0.0202 (7)	0.0022 (6)	0.0016 (6)	−0.0012 (6)
C20	0.0201 (7)	0.0278 (7)	0.0215 (7)	0.0003 (5)	0.0046 (5)	−0.0005 (5)
C21	0.0329 (9)	0.0517 (10)	0.0268 (8)	0.0055 (7)	0.0081 (7)	0.0003 (7)
C22	0.0394 (10)	0.0496 (10)	0.0305 (9)	0.0007 (8)	0.0117 (7)	−0.0062 (7)
C23	0.0316 (8)	0.0454 (10)	0.0329 (9)	0.0093 (7)	0.0104 (7)	0.0043 (7)
C24	0.0257 (8)	0.0432 (9)	0.0309 (8)	0.0016 (7)	0.0082 (6)	−0.0037 (7)
C25	0.0295 (8)	0.0323 (8)	0.0335 (8)	0.0039 (6)	0.0088 (6)	0.0062 (6)
C26	0.0326 (9)	0.0393 (9)	0.0503 (11)	0.0119 (7)	0.0040 (8)	0.0043 (8)
C27	0.0297 (9)	0.0549 (11)	0.0465 (11)	0.0148 (8)	0.0086 (8)	−0.0015 (9)
C28	0.0525 (11)	0.0468 (10)	0.0426 (10)	0.0168 (9)	0.0246 (9)	0.0038 (8)
N1	0.0253 (7)	0.0422 (8)	0.0356 (8)	0.0100 (6)	0.0036 (6)	−0.0001 (6)
N2	0.0209 (6)	0.0323 (6)	0.0181 (6)	0.0041 (5)	0.0031 (4)	0.0015 (5)
N3	0.0203 (6)	0.0325 (6)	0.0186 (6)	0.0036 (5)	0.0055 (5)	−0.0002 (5)
N4	0.0210 (6)	0.0328 (6)	0.0194 (6)	0.0029 (5)	0.0038 (5)	0.0026 (5)
N5	0.0192 (6)	0.0324 (6)	0.0175 (6)	0.0023 (5)	0.0027 (5)	0.0002 (5)
N6	0.0228 (6)	0.0338 (7)	0.0245 (6)	0.0012 (5)	0.0063 (5)	−0.0025 (5)
N7	0.0331 (7)	0.0363 (7)	0.0312 (7)	0.0081 (6)	0.0152 (6)	0.0017 (6)
O1	0.0325 (6)	0.0502 (7)	0.0213 (6)	0.0055 (5)	0.0077 (5)	0.0010 (5)

### N,N,3-Trimethyl-4-[6-(4-methylpiperazin-1-yl)-1H,3'H-[2,5'-bibenzo[d]imidazol]-2'-yl]aniline monohydrate (1_hydrate) Geometric parameters (Å, º)

**Table:**

C1—C6	1.407 (2)	C19—C20	1.390 (2)
C1—C2	1.412 (2)	C19—H19	0.9300
C1—C7	1.468 (2)	C20—N5	1.3783 (19)
C2—C3	1.387 (2)	C21—N6	1.446 (2)
C2—C25	1.509 (2)	C21—C22	1.518 (2)
C3—C4	1.409 (2)	C21—H21A	0.9700
C3—H3	0.9300	C21—H21B	0.9700
C4—N1	1.374 (2)	C22—N7	1.453 (2)
C4—C5	1.411 (2)	C22—H22A	0.9700
C5—C6	1.383 (2)	C22—H22B	0.9700
C5—H5	0.9300	C23—N7	1.467 (2)
C6—H6	0.9300	C23—C24	1.509 (2)
C7—N2	1.3344 (19)	C23—H23A	0.9700
C7—N3	1.3687 (19)	C23—H23B	0.9700
C8—N2	1.3899 (19)	C24—N6	1.465 (2)
C8—C9	1.395 (2)	C24—H24A	0.9700
C8—C13	1.405 (2)	C24—H24B	0.9700
C9—C10	1.395 (2)	C25—H25A	0.9600
C9—H9	0.9300	C25—H25B	0.9600
C10—C11	1.413 (2)	C25—H25C	0.9600
C10—C14	1.464 (2)	C26—N1	1.451 (2)
C11—C12	1.384 (2)	C26—H26A	0.9600
C11—H11	0.9300	C26—H26B	0.9600
C12—C13	1.394 (2)	C26—H26C	0.9600
C12—H12	0.9300	C27—N1	1.449 (2)
C13—N3	1.3712 (19)	C27—H27A	0.9600
C14—N4	1.3286 (19)	C27—H27B	0.9600
C14—N5	1.3691 (19)	C27—H27C	0.9600
C15—N4	1.3936 (19)	C28—N7	1.458 (2)
C15—C16	1.400 (2)	C28—H28A	0.9600
C15—C20	1.402 (2)	C28—H28B	0.9600
C16—C17	1.395 (2)	C28—H28C	0.9600
C16—H16	0.9300	N3—H3A	0.93 (2)
C17—C18	1.417 (2)	N5—H5A	0.89 (2)
C17—N6	1.4190 (19)	O1—H1A	0.93 (4)
C18—C19	1.374 (2)	O1—H1B	0.95 (3)
C18—H18	0.9300		
			
C6—C1—C2	117.89 (14)	N6—C21—H21B	109.6
C6—C1—C7	118.69 (14)	C22—C21—H21B	109.6
C2—C1—C7	123.34 (13)	H21A—C21—H21B	108.1
C3—C2—C1	119.18 (14)	N7—C22—C21	111.93 (15)
C3—C2—C25	117.50 (14)	N7—C22—H22A	109.2
C1—C2—C25	123.31 (13)	C21—C22—H22A	109.2
C2—C3—C4	123.14 (15)	N7—C22—H22B	109.2
C2—C3—H3	118.4	C21—C22—H22B	109.2
C4—C3—H3	118.4	H22A—C22—H22B	107.9
N1—C4—C3	120.91 (15)	N7—C23—C24	110.73 (14)
N1—C4—C5	122.05 (15)	N7—C23—H23A	109.5
C3—C4—C5	117.02 (14)	C24—C23—H23A	109.5
C6—C5—C4	120.18 (15)	N7—C23—H23B	109.5
C6—C5—H5	119.9	C24—C23—H23B	109.5
C4—C5—H5	119.9	H23A—C23—H23B	108.1
C5—C6—C1	122.42 (15)	N6—C24—C23	110.86 (14)
C5—C6—H6	118.8	N6—C24—H24A	109.5
C1—C6—H6	118.8	C23—C24—H24A	109.5
N2—C7—N3	112.50 (13)	N6—C24—H24B	109.5
N2—C7—C1	126.94 (13)	C23—C24—H24B	109.5
N3—C7—C1	120.53 (13)	H24A—C24—H24B	108.1
N2—C8—C9	129.60 (13)	C2—C25—H25A	109.5
N2—C8—C13	110.06 (12)	C2—C25—H25B	109.5
C9—C8—C13	120.34 (13)	H25A—C25—H25B	109.5
C10—C9—C8	117.80 (13)	C2—C25—H25C	109.5
C10—C9—H9	121.1	H25A—C25—H25C	109.5
C8—C9—H9	121.1	H25B—C25—H25C	109.5
C9—C10—C11	120.75 (13)	N1—C26—H26A	109.5
C9—C10—C14	119.36 (13)	N1—C26—H26B	109.5
C11—C10—C14	119.85 (13)	H26A—C26—H26B	109.5
C12—C11—C10	122.05 (13)	N1—C26—H26C	109.5
C12—C11—H11	119.0	H26A—C26—H26C	109.5
C10—C11—H11	119.0	H26B—C26—H26C	109.5
C11—C12—C13	116.47 (13)	N1—C27—H27A	109.5
C11—C12—H12	121.8	N1—C27—H27B	109.5
C13—C12—H12	121.8	H27A—C27—H27B	109.5
N3—C13—C12	131.98 (14)	N1—C27—H27C	109.5
N3—C13—C8	105.46 (12)	H27A—C27—H27C	109.5
C12—C13—C8	122.57 (13)	H27B—C27—H27C	109.5
N4—C14—N5	112.69 (13)	N7—C28—H28A	109.5
N4—C14—C10	124.72 (13)	N7—C28—H28B	109.5
N5—C14—C10	122.58 (13)	H28A—C28—H28B	109.5
N4—C15—C16	129.68 (13)	N7—C28—H28C	109.5
N4—C15—C20	109.67 (13)	H28A—C28—H28C	109.5
C16—C15—C20	120.64 (13)	H28B—C28—H28C	109.5
C17—C16—C15	118.43 (14)	C4—N1—C27	120.55 (15)
C17—C16—H16	120.8	C4—N1—C26	119.35 (15)
C15—C16—H16	120.8	C27—N1—C26	119.08 (14)
C16—C17—C18	119.50 (14)	C7—N2—C8	104.57 (12)
C16—C17—N6	122.47 (13)	C7—N3—C13	107.39 (12)
C18—C17—N6	117.92 (13)	C7—N3—H3A	130.9 (14)
C19—C18—C17	122.29 (14)	C13—N3—H3A	121.5 (14)
C19—C18—H18	118.9	C14—N4—C15	104.91 (12)
C17—C18—H18	118.9	C14—N5—C20	106.94 (12)
C18—C19—C20	117.78 (14)	C14—N5—H5A	126.4 (13)
C18—C19—H19	121.1	C20—N5—H5A	126.0 (13)
C20—C19—H19	121.1	C17—N6—C21	117.43 (13)
N5—C20—C19	132.90 (14)	C17—N6—C24	115.25 (13)
N5—C20—C15	105.78 (12)	C21—N6—C24	109.98 (13)
C19—C20—C15	121.32 (14)	C22—N7—C28	110.09 (16)
N6—C21—C22	110.31 (15)	C22—N7—C23	109.16 (14)
N6—C21—H21A	109.6	C28—N7—C23	110.83 (14)
C22—C21—H21A	109.6	H1A—O1—H1B	102 (3)
			
C6—C1—C2—C3	3.1 (2)	C18—C19—C20—N5	178.45 (16)
C7—C1—C2—C3	−173.56 (13)	C18—C19—C20—C15	−1.8 (2)
C6—C1—C2—C25	−177.96 (14)	N4—C15—C20—N5	−0.14 (16)
C7—C1—C2—C25	5.4 (2)	C16—C15—C20—N5	−178.90 (14)
C1—C2—C3—C4	0.2 (2)	N4—C15—C20—C19	−179.98 (14)
C25—C2—C3—C4	−178.84 (14)	C16—C15—C20—C19	1.3 (2)
C2—C3—C4—N1	177.91 (14)	N6—C21—C22—N7	−57.8 (2)
C2—C3—C4—C5	−3.5 (2)	N7—C23—C24—N6	58.11 (19)
N1—C4—C5—C6	−177.94 (15)	C3—C4—N1—C27	−170.86 (16)
C3—C4—C5—C6	3.5 (2)	C5—C4—N1—C27	10.6 (2)
C4—C5—C6—C1	−0.3 (2)	C3—C4—N1—C26	−2.5 (2)
C2—C1—C6—C5	−3.1 (2)	C5—C4—N1—C26	178.98 (16)
C7—C1—C6—C5	173.73 (14)	N3—C7—N2—C8	−1.61 (17)
C6—C1—C7—N2	159.76 (15)	C1—C7—N2—C8	176.41 (14)
C2—C1—C7—N2	−23.6 (2)	C9—C8—N2—C7	−178.69 (15)
C6—C1—C7—N3	−22.4 (2)	C13—C8—N2—C7	1.39 (17)
C2—C1—C7—N3	154.23 (14)	N2—C7—N3—C13	1.24 (17)
N2—C8—C9—C10	−179.95 (15)	C1—C7—N3—C13	−176.92 (13)
C13—C8—C9—C10	0.0 (2)	C12—C13—N3—C7	179.70 (16)
C8—C9—C10—C11	−0.1 (2)	C8—C13—N3—C7	−0.30 (16)
C8—C9—C10—C14	177.48 (13)	N5—C14—N4—C15	0.15 (17)
C9—C10—C11—C12	0.9 (2)	C10—C14—N4—C15	−178.70 (14)
C14—C10—C11—C12	−176.70 (14)	C16—C15—N4—C14	178.62 (16)
C10—C11—C12—C13	−1.4 (2)	C20—C15—N4—C14	0.00 (16)
C11—C12—C13—N3	−178.69 (15)	N4—C14—N5—C20	−0.24 (17)
C11—C12—C13—C8	1.3 (2)	C10—C14—N5—C20	178.64 (13)
N2—C8—C13—N3	−0.68 (17)	C19—C20—N5—C14	−179.96 (16)
C9—C8—C13—N3	179.39 (13)	C15—C20—N5—C14	0.22 (16)
N2—C8—C13—C12	179.32 (14)	C16—C17—N6—C21	3.4 (2)
C9—C8—C13—C12	−0.6 (2)	C18—C17—N6—C21	179.43 (15)
C9—C10—C14—N4	−19.8 (2)	C16—C17—N6—C24	135.47 (16)
C11—C10—C14—N4	157.83 (15)	C18—C17—N6—C24	−48.47 (19)
C9—C10—C14—N5	161.50 (14)	C22—C21—N6—C17	−168.74 (14)
C11—C10—C14—N5	−20.9 (2)	C22—C21—N6—C24	56.83 (19)
N4—C15—C16—C17	−177.97 (14)	C23—C24—N6—C17	166.65 (13)
C20—C15—C16—C17	0.5 (2)	C23—C24—N6—C21	−57.84 (18)
C15—C16—C17—C18	−1.7 (2)	C21—C22—N7—C28	178.82 (15)
C15—C16—C17—N6	174.29 (14)	C21—C22—N7—C23	56.96 (19)
C16—C17—C18—C19	1.2 (2)	C24—C23—N7—C22	−56.93 (19)
N6—C17—C18—C19	−174.97 (15)	C24—C23—N7—C28	−178.34 (16)
C17—C18—C19—C20	0.5 (2)		

### N,N,3-Trimethyl-4-[6-(4-methylpiperazin-1-yl)-1H,3'H-[2,5'-bibenzo[d]imidazol]-2'-yl]aniline monohydrate (1_hydrate) Hydrogen-bond geometry (Å, º)

**Table:**

*D*—H···*A*	*D*—H	H···*A*	*D*···*A*	*D*—H···*A*
O1—H1*A*···N7^i^	0.93 (4)	1.93 (4)	2.858 (2)	177 (3)
O1—H1*B*···N4^ii^	0.95 (3)	1.94 (3)	2.8905 (18)	177 (2)
N3—H3*A*···O1	0.93 (2)	1.82 (2)	2.7338 (17)	170 (2)
N5—H5*A*···N2^iii^	0.89 (2)	2.15 (2)	3.0199 (18)	167.2 (18)
O1—H1*A*···N7^i^	0.93 (4)	1.93 (4)	2.858 (2)	177 (3)
O1—H1*B*···N4^ii^	0.95 (3)	1.94 (3)	2.8905 (18)	177 (2)
N3—H3*A*···O1	0.93 (2)	1.82 (2)	2.7338 (17)	170 (2)
N5—H5*A*···N2^iii^	0.89 (2)	2.15 (2)	3.0199 (18)	167.2 (18)

Symmetry codes: (i) *x*−3/2, −*y*+1/2, *z*+1/2; (ii) *x*−1/2, −*y*+1/2, *z*+1/2; (iii) *x*+1/2, −*y*+1/2, *z*+1/2.
